# Supplementary material for: Fe3O4-doped mesoporous carbon cathode with a plumber’s nightmare structure for high-performance Li-S batteries
Source: Nat Commun. 2024 Jun 27;15:5451. doi: 10.1038/s41467-024-49826-5 (PMC11211388; doi:10.1038/s41467-024-49826-5)
Supplement: Supplementary file 1 — Supplementary Information [file 41467_2024_49826_MOESM1_ESM.pdf]

Supplementary Information

Fe_3O_4-doped mesoporous carbon cathode with a plumber's nightmare structure for high-performance Li-S batteries

Han Zhang[1,2], Mengtian Zhang[3], Ruiyi Liu[1,2], Tengfeng He[4], Luoxing Xiang[1,2], Xinru Wu[3], Zhihong Piao[3], Yeyang Jia[3], Chongyin Zhang[4], Hong Li*[3], Fugui Xu*[1,2], Guangmin Zhou*[3], and Yiyong Mai*[1,2]

[1] School of Chemistry and Chemical Engineering, Shanghai Jiao Tong University, 800 Dongchuan Road, Shanghai 200240, China

[2] Frontiers Science Center for Transformative Molecules, Shanghai Jiao Tong University, 800 Dongchuan Road, Shanghai 200240, China

[3] Tsinghua-Berkeley Shenzhen Institute & Tsinghua Shenzhen International Graduate School, Tsinghua University, Shenzhen 518055, China

[4] Shanghai Aerospace Equipments Manufacturer Co., Ltd., 100 Huaning Road, Shanghai 200245, China

*Corresponding author: Hong Li, e-mail: melihong@alumni.sjtu.edu.cn; Fugui Xu, e-mail: xufg1227@sjtu.edu.cn; Guangmin Zhou, e-mail: guangminzhou@sz.tsinghua.edu.cn; Yiyong Mai, e-mail: mai@sjtu.edu.cn.

Supplementary Figures

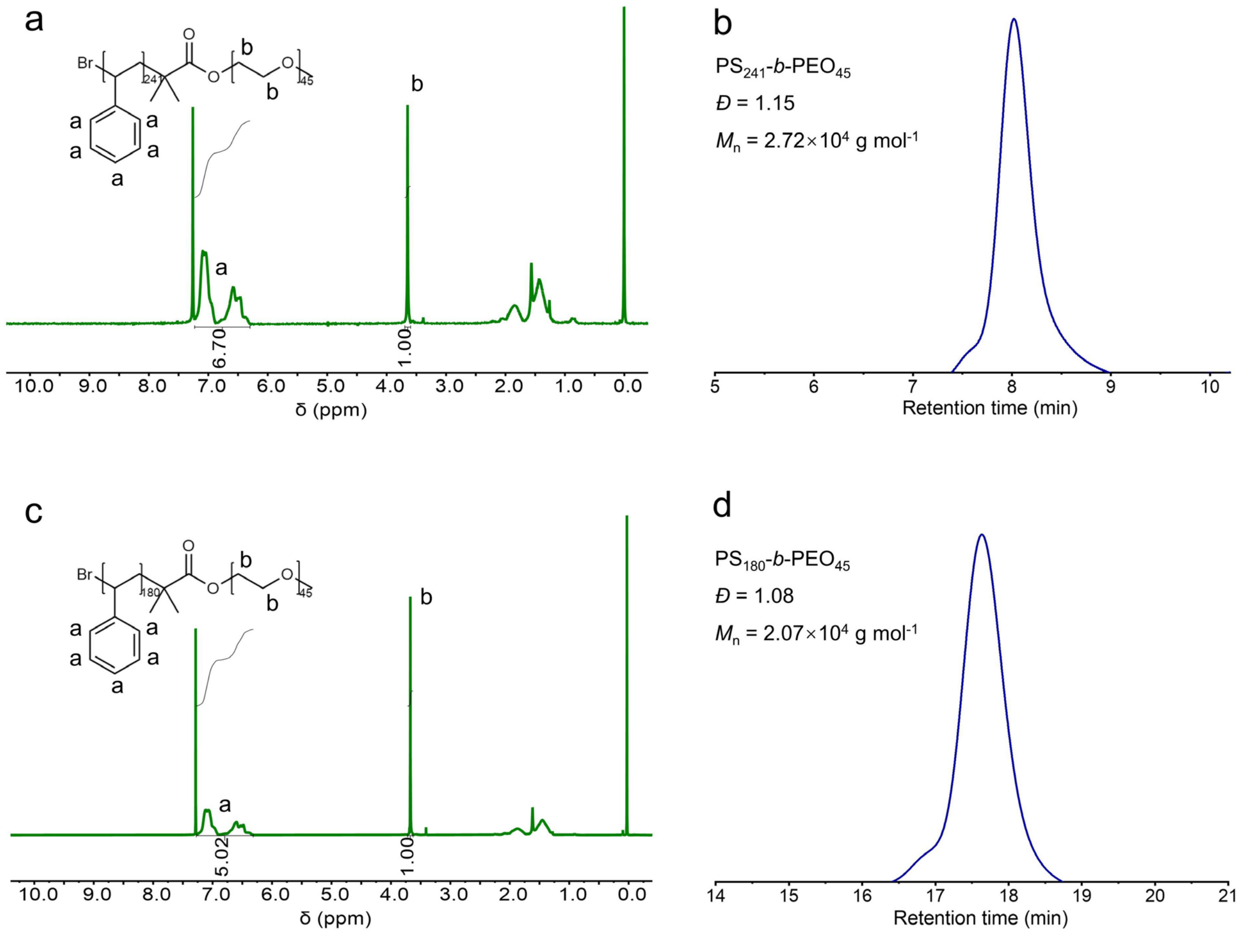

Supplementary Fig. 1. Characterizations of PS-*b*-PEO: a ^{1}H-NMR spectrum of PS_{241}-*b*-PEO_{45} (The inset is the molecular formula of PS_{241}-*b*-PEO_{45}). **b** GPC trace of PS_{241}-*b*-PEO_{45} (The inset is the molecular weight information of PS_{241}-*b*-PEO_{45}). **c** ^{1}H-NMR spectrum of PS_{180}-*b*-PEO_{45} (The inset is the molecular formula of PS_{180}-*b*-PEO_{45}). **d** GPC trace of PS_{180}-*b*-PEO_{45} (The inset is the molecular weight information of PS_{180}-*b*-PEO_{45}). The degrees of polymerization of the PS block were calculated according to the NMR results, which are closed to those obtained from GPC.

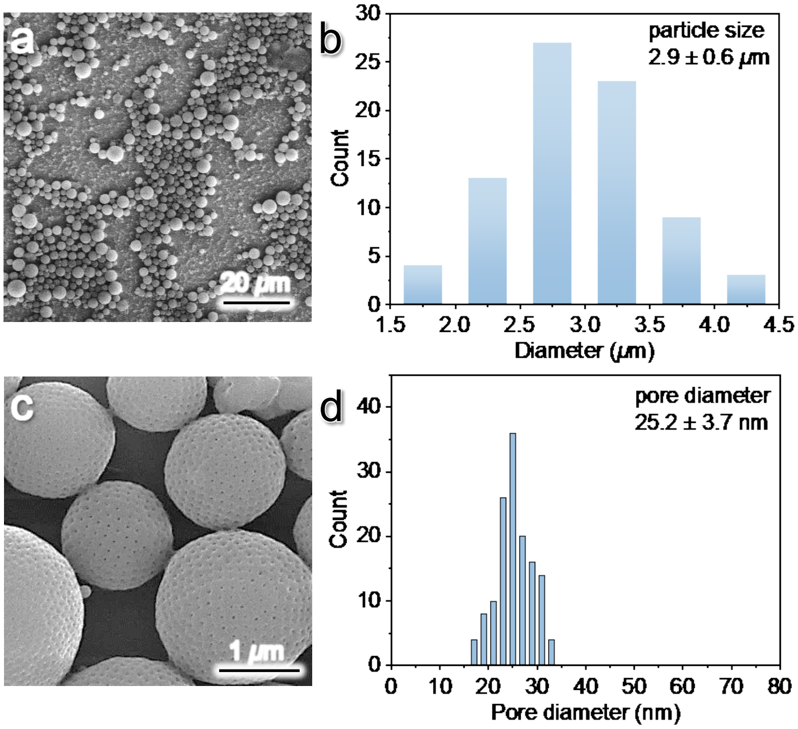

Supplementary Fig. 2. Morphologies and structural parameters of DP-PC: a Low-magnification regular SEM image of DP-PC. **b** Size distribution histogram. The average particle size of DP-PC by statistical analysis of 200 particles in the SEM images is 2.9 ± 0.6 μm. **c** High-magnification SEM image of DP-PC. **d** Pore diameter distribution of DP-PC. The average pore diameter of DP-PC by statistics is 25.2 ± 3.7 nm.

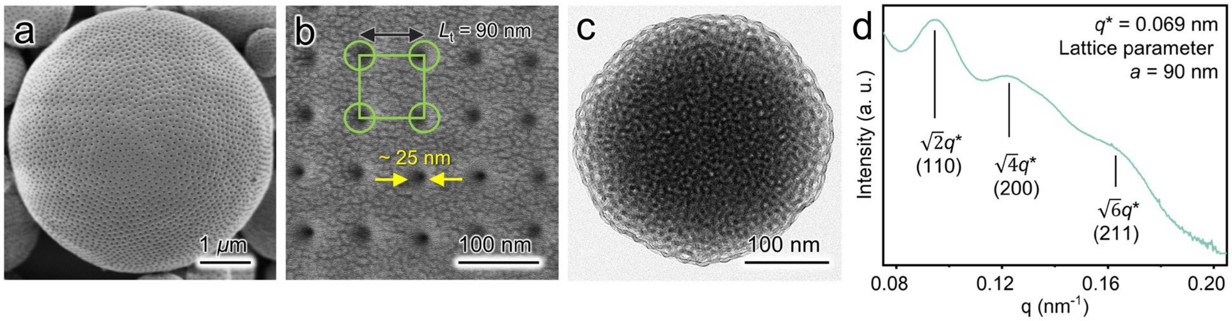

Supplementary Fig. 3. Characterizations of DP-PC: a High-magnification SEM image of a DP-PC particle. **b** High magnification SEM image of the particle surface. The green framework stresses a unit cell consisting of open mesopores. L_t means the length of a unit cell on the PC surface. **c** A typical TEM image showing the internal porous structure of a PC along the (100) direction. **d** SAXS pattern of the dried PC powder, lattice parameter $a = 2\pi/q^*$. These results demonstrate that DP-PCs had a double primitive structure ($Im\bar{3}m$ symmetry) with a set of open mesochannels.[1]

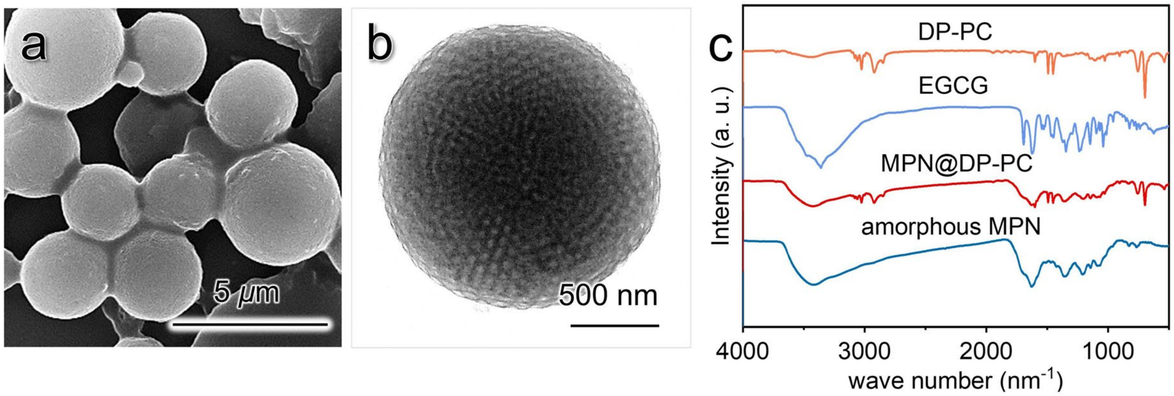

Supplementary Fig. 4. Characterizations of MPN@DP-PC: a SEM image of MPN@DP-PC. **b** TEM image of MPN@DP-PC. **c** FTIR spectra of DP-PC, EGCG, MPN@DP-PC, and amorphous MPN.

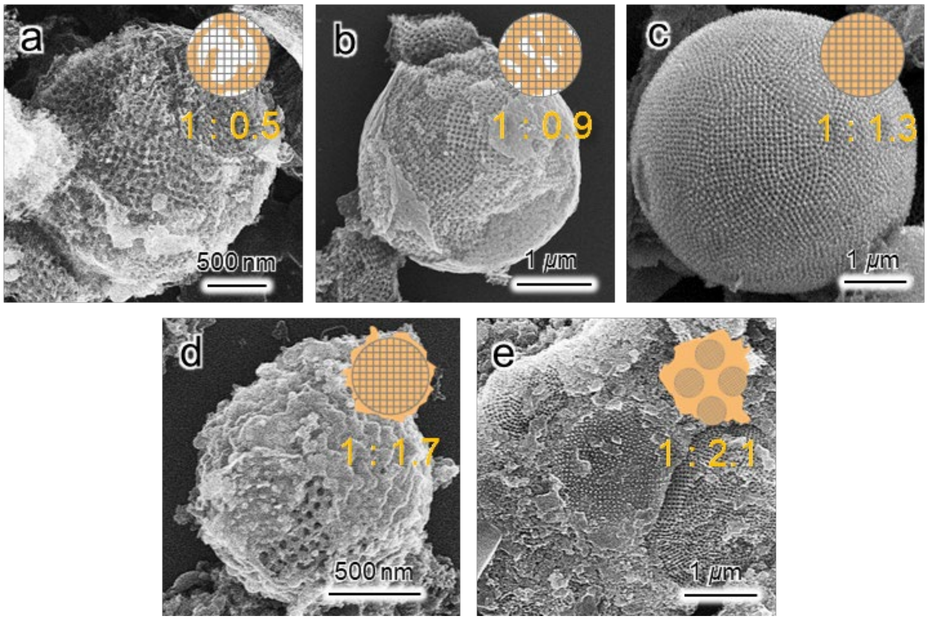

Supplementary Fig. 5. The SEM images of the pyrolyzed carbon samples prepared with different EGCG/Fe^{3+} molar ratio: a 1:0.5, **b** 1:0.9, **c** 1:1.3, **d** 1:1.7 and **e** 1:2.1. When the molar ratio of n_{EGCG} / $n_{Fe^{3+}}$ < 1.3 (n_{EGCG} / $n_{Fe^{3+}}$ = 1:0.5 and 1:0.9), the interior of the template was incompletely replicated, forming fragments or collapsed carbon spheres after pyrolysis (**a, b**). Further increasing the ratio to 1:1.3, the PC template was fully filled, and intact SP-Fe_3O_4-C could be obtained (**c**). Once the ratio is larger than 1.3 (n_{EGCG} / $n_{Fe^{3+}}$ = 1:1.7 and 1:2.1), the precursors could fill the template while the excess precursors outside the template formed agglomerates on the surfaces (**d, e**). With the increase of $n_{Fe^{3+}}$, the proportion of the random agglomerates increased accordingly. (The insets are the models of SP-Fe_3O_4-C with different EGCG/Fe^{3+} molar ratios.)

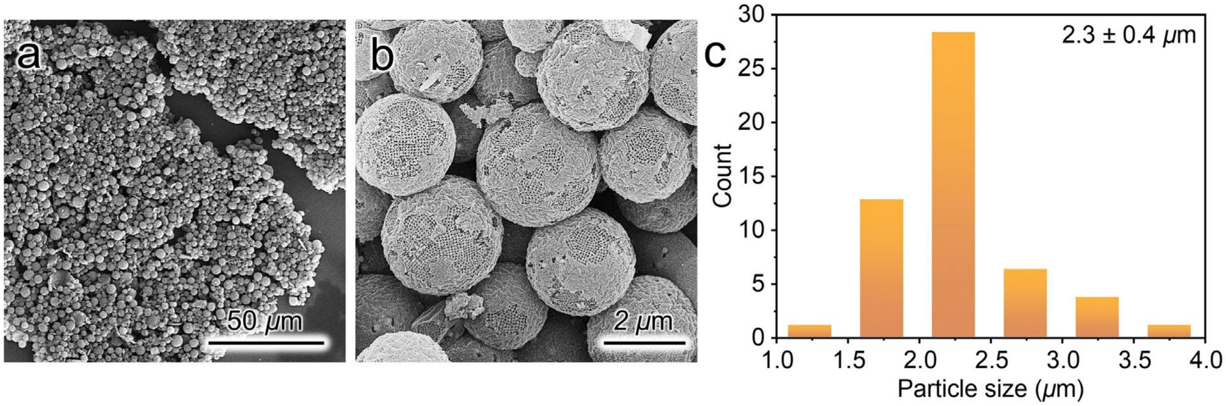

Supplementary Fig. 6. Morphology and particle size statistic of SP-Fe_3O_4-C: a, b A low-magnification SEM image of SP-Fe_3O_4-C in large areas. **c** Particle size distribution histogram and the average diameter (d = 2.3 ± 0.4 μm) of SP-Fe_3O_4-C.

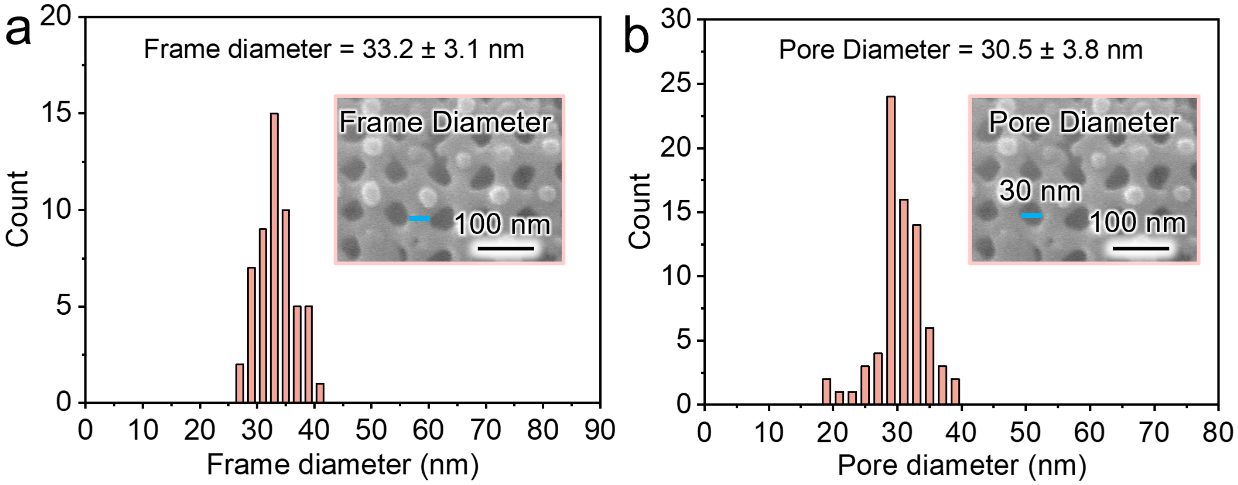

Supplementary Fig. 7. Statistics of frame and pore size in SP-Fe_3O_4-C: a Frame diameter distribution histogram and the average frame diameter of SP-Fe_3O_4-C (The inset is the enlarged view of the SP-Fe_3O_4-C surface, the blue line means the frame diameter of SP-Fe_3O_4-C). **b** Pore diameter distribution histogram and the average pore diameter of SP-Fe_3O_4-C. (The inset is the enlarged view of the SP-Fe_3O_4-C surface and the blue line means the pore diameter of SP-Fe_3O_4-C).

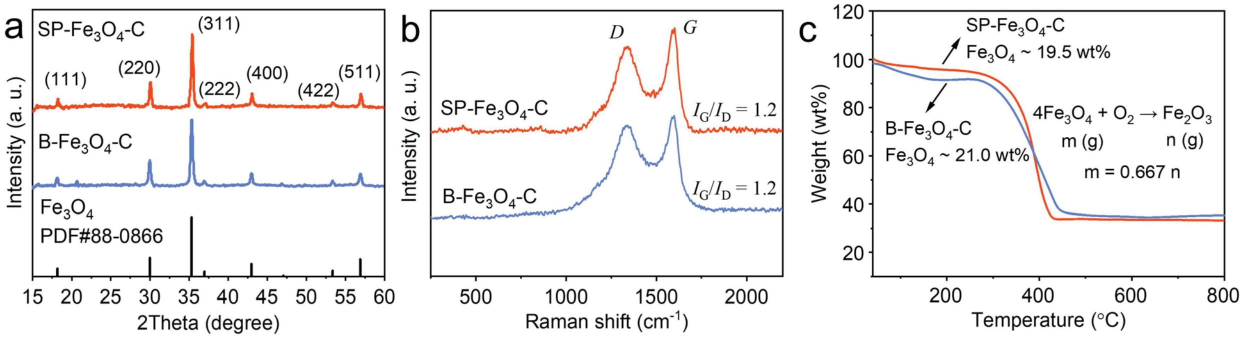

Supplementary Fig. 8. XRD, Raman and TGA of SP-Fe_3O_4-C and B-Fe_3O_4-C: a X-Ray Diffraction (XRD) analysis patterns of SP-Fe_3O_4-C, B-Fe_3O_4-C and the standard PDF card of Fe_3O_4 (PDF#88-0866); only the peaks of Fe_3O_4 are observed in the XRD patterns, while the two broad peaks ($2\theta = 25°$ and $43°$) attributed to carbon are overlaid by the strong signals of Fe_3O_4;[2,3] yet the carbon signals appeared after the removed of Fe_3O_4 by acid etching (Supplementary Fig. 18a). **b** Raman spectra of SP-Fe_3O_4-C and B-Fe_3O_4-C. **c** Thermal gravimetric analysis (TGA) curves of SP-Fe_3O_4-C and B-Fe_3O_4-C under the air atmosphere. During the heating process (heating from the room temperature to 800 °C under the air atmosphere), Fe_3O_4 was completely oxidized to Fe_2O_3, leading to a slight weight increase in the whole weight loss. After deducting the oxygen increase from the whole weight loss, the contents of Fe_3O_4 and carbon of SP-Fe_3O_4-C were calculated to be 19.5 wt% and 81.5 wt%, respectively; the contents of Fe_3O_4 and carbon of B-Fe_3O_4-C were calculated to be 21.0 wt% and 79.0 wt%, respectively.

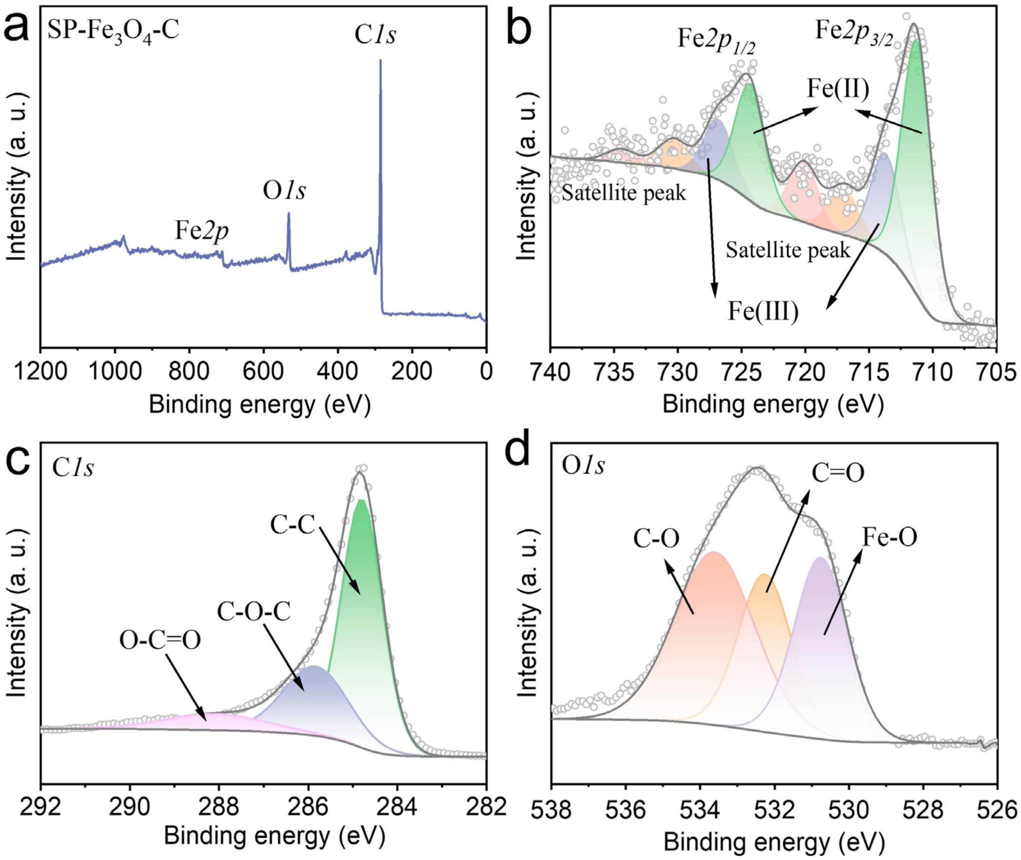

Supplementary Fig. 9. XPS spectra of SP-Fe_3O_4-C: a XPS survey. **b** High-resolution Fe *2p* spectrum. **c** High-resolution C*1s* spectrum. **d** High-resolution O*1s* spectrum.

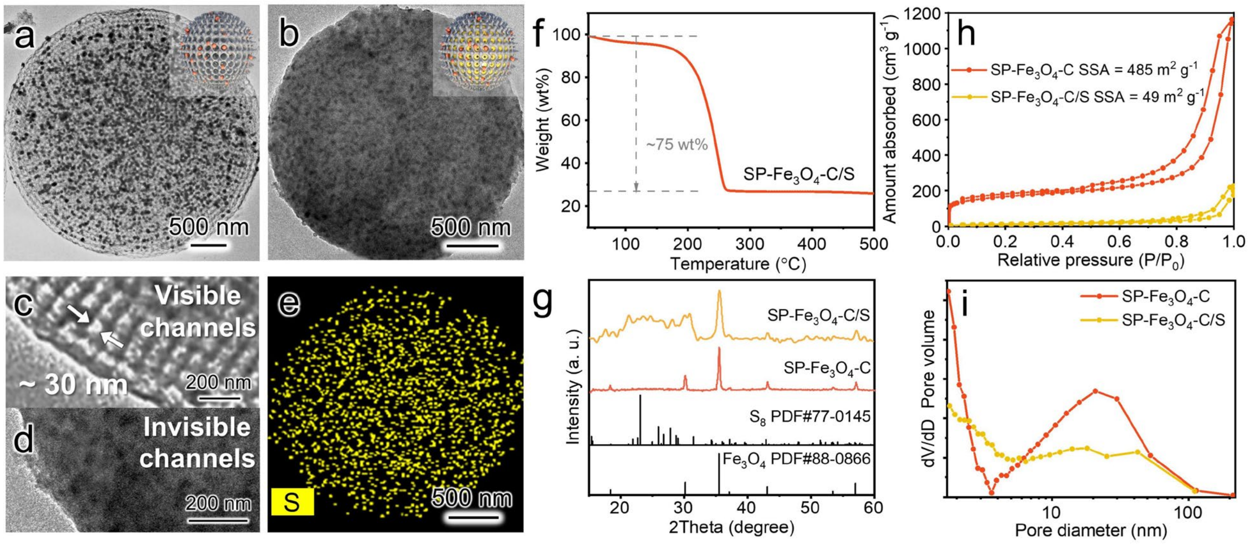

Supplementary Fig. 10. Successful sulfur loading in SP-Fe_3O_4-C: a TEM image of a SP-Fe_3O_4-C particle (the inset is a model of SP-Fe_3O_4-C). **b** TEM image of a SP-Fe_3O_4-C/S particle (the inset is a model of SP-Fe_3O_4-C/S). **c** Magnified view of SP-Fe_3O_4-C (clear open channels are observed in SP-Fe_3O_4-C). **d** Magnified view of SP-Fe_3O_4-C/S (open pores are invisible). **e** EDS mapping image (Sulfur element) of SP-Fe_3O_4-C/S. **f** TGA curve of SP-Fe_3O_4-C/S. **g** X-ray diffraction (XRD) patterns of SP-Fe_3O_4-C/S and SP-Fe_3O_4-C. **h** N_2 adsorption–desorption isotherm of SP-Fe_3O_4-C and SP-Fe_3O_4-C/S. **i** Pore size distribution of SP-Fe_3O_4-C and SP-Fe_3O_4-C/S.

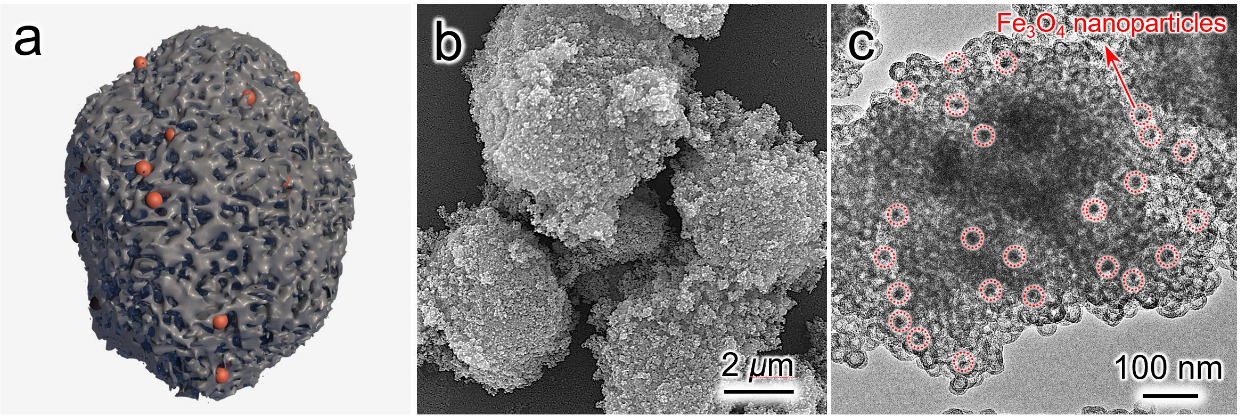

Supplementary Fig. 11. Morphology of B-Fe_3O_4-C: a Schematic illustration of B-Fe_3O_4-C. **b** Low-magnification SEM image of B-Fe_3O_4-C. **c** TEM image of B-Fe_3O_4-C.

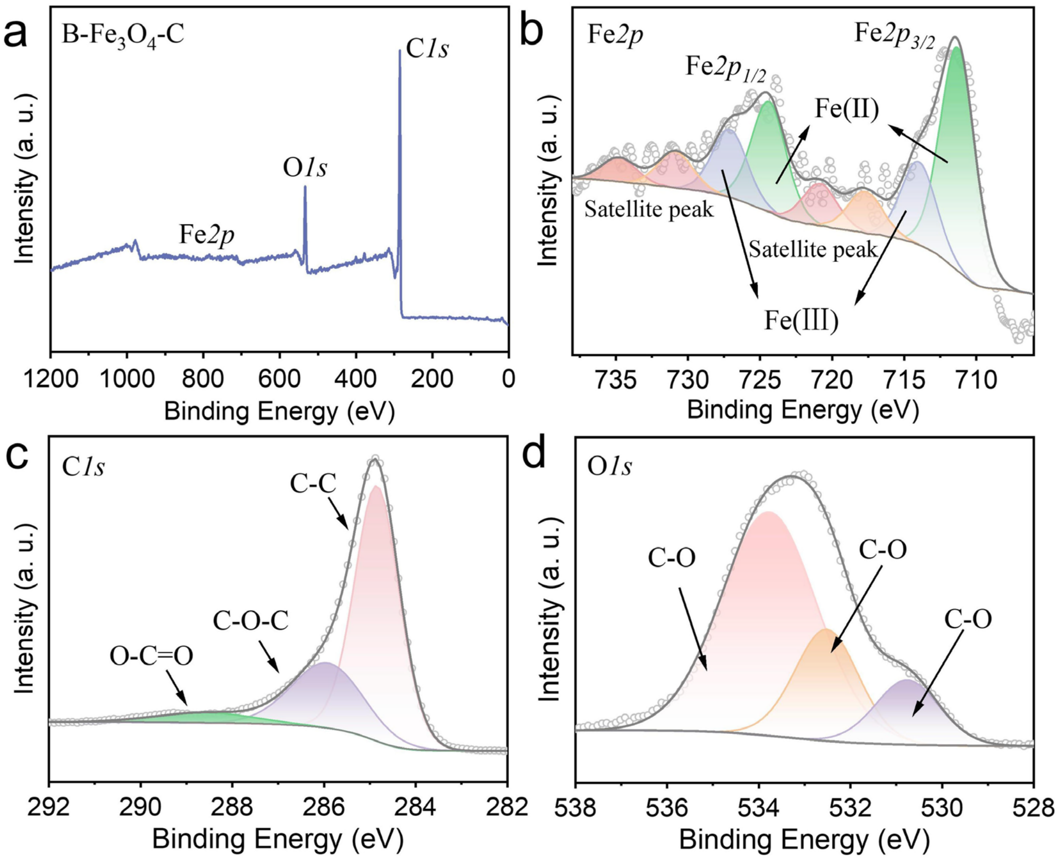

Supplementary Fig. 12. XPS spectra of B-Fe_3O_4-C: a survey. **b** High-resolution Fe *2p* spectrum. **c** High-resolution C*1s* spectrum. **d** High-resolution O*1s* spectrum.

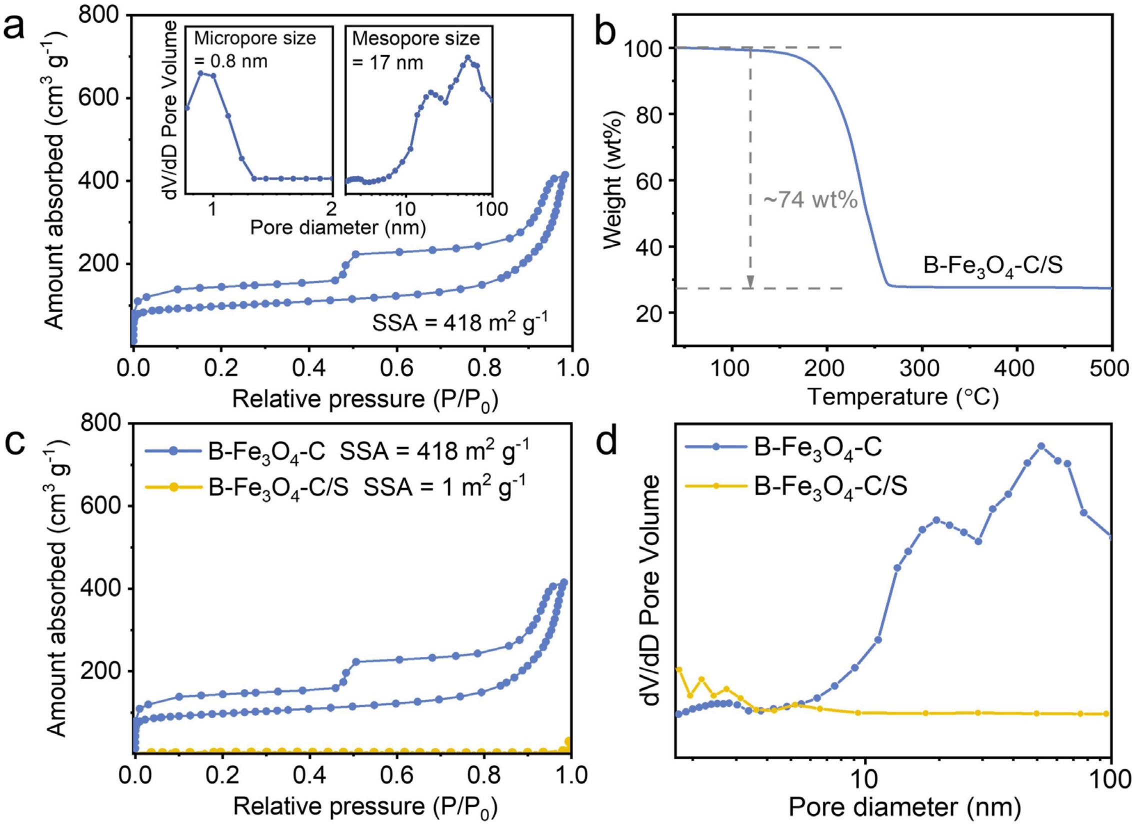

Supplementary Fig. 13. Successful sulfur loading in B-Fe_3O_4-C/S: a Nitrogen adsorption isotherm and pore size distribution of B-Fe_3O_4-C (inset). **b** Thermogravimetric analysis curve of the B-Fe_3O_4-C/S. **c** N_2 adsorption–desorption isotherm of B-Fe_3O_4-C and B-Fe_3O_4-C/S. **d** Pore distribution of B-Fe_3O_4-C and B-Fe_3O_4-C/S.

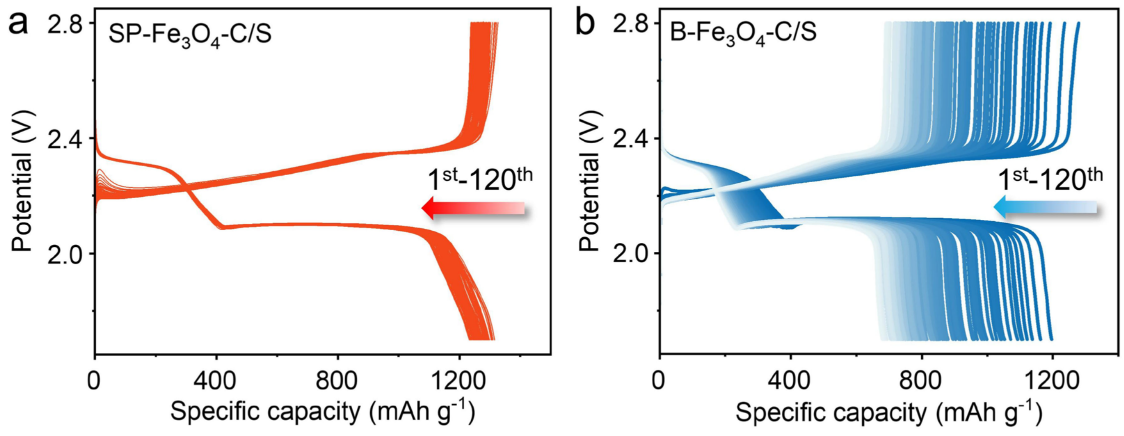

Supplementary Fig. 14. Charge/discharge curves at different cycles at 0.2 C: a SP-Fe_3O_4-C/S; **b** B-Fe_3O_4-C/S.

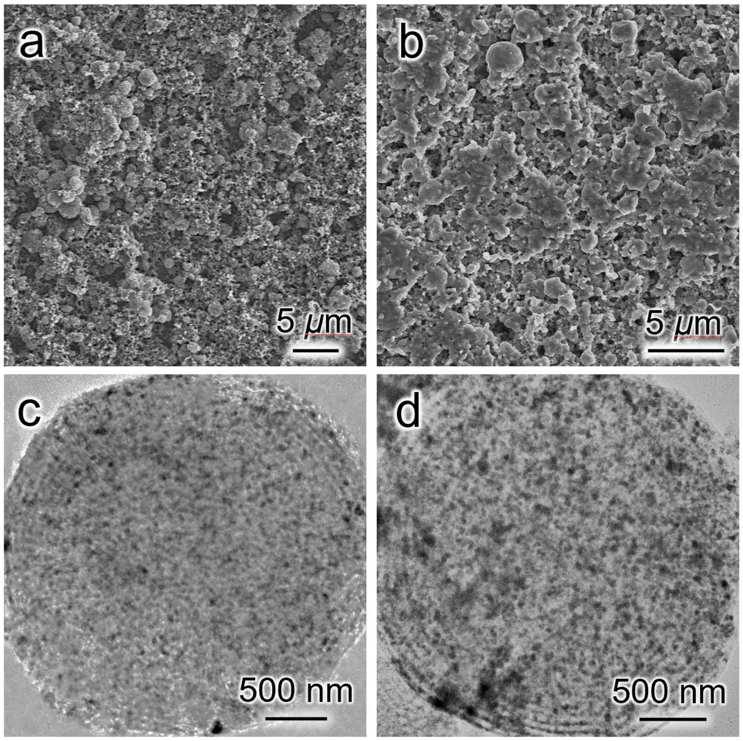

Supplementary Fig. 15. Morphology change of SP-Fe_3O_4-C/S cathode before/after cycle: a SEM image of the SP-Fe_3O_4-C/S cathode before cycle. **b** SEM image of the SP-Fe_3O_4-C/S cathode after 120 cycles at 0.2 C. **c** TEM image of a SP-Fe_3O_4-C/S particle before cycle. **d** TEM image of a SP-Fe_3O_4-C/S particle after cycling at 0.2 C for 120 cycles; the impurities consisted of residual carbon black particles, PVDF binder and electrolyte salts, which could not be removed completely.

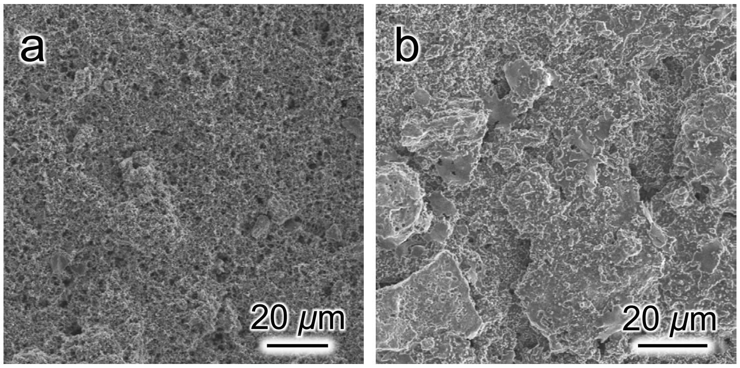

Supplementary Fig. 16. Morphology change of B-Fe_3O_4-C/S cathode before/after cycle: a The SEM image of the B-Fe_3O_4-C/S cathode before cycle. **b** The SEM image of the B-Fe_3O_4-C/S cathode after 120 cycles at 0.2 C. The structure of the B-Fe_3O_4-C/S cathode underwent significant changes after multiple charge and discharge cycles, and bulky sulfur aggregates appeared.

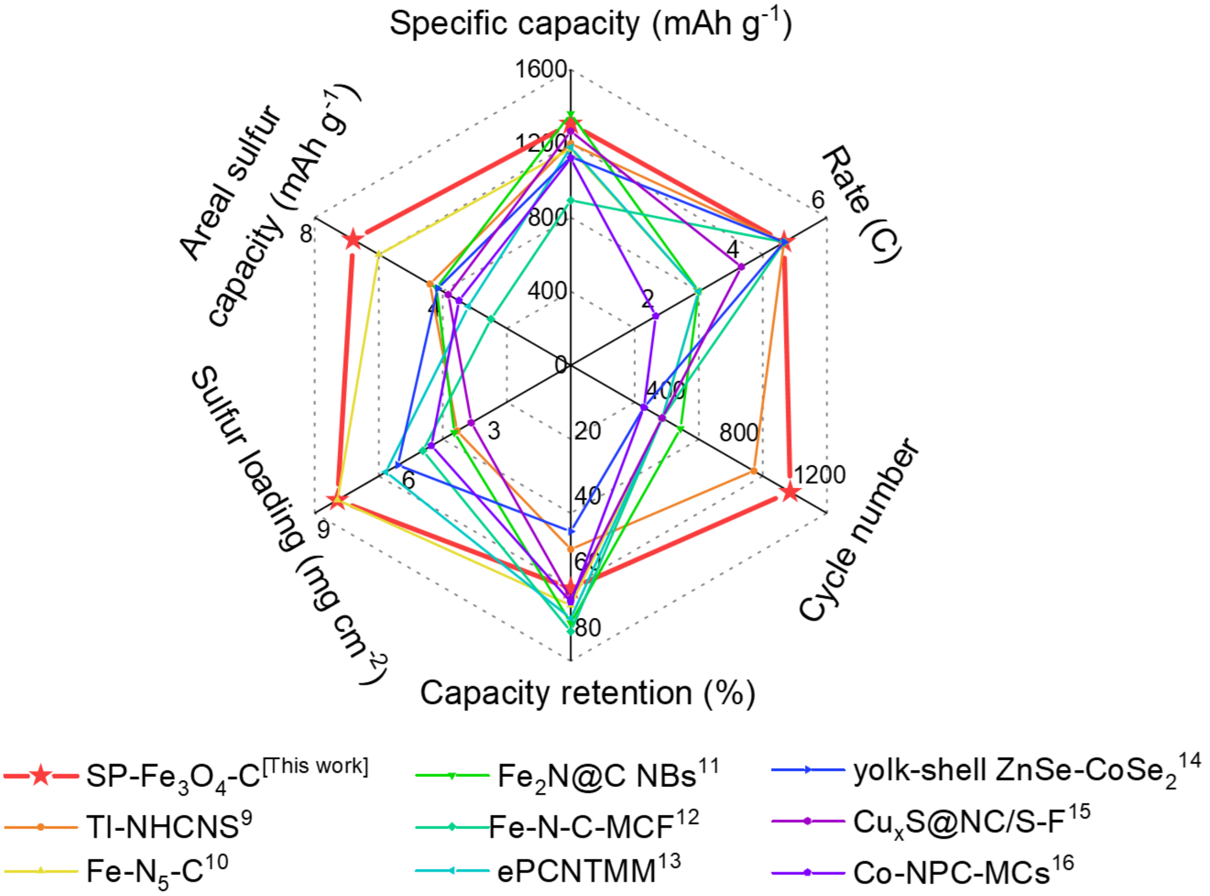

Supplementary Fig. 17. A performance radar chart of the Li-S battery with the SP-Fe_3O_4-C/S cathode in comparison with the reported Li-S batteries with representative carbon-based cathodes within the last five years.[4-11]

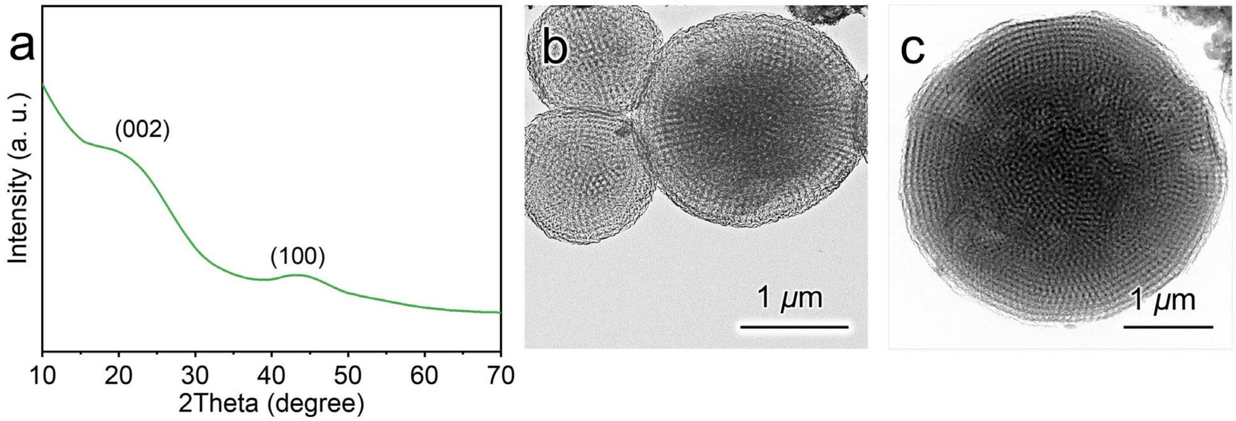

Supplementary Fig. 18. Characterizations of SP-C: a XRD spectrum of SP-C. **b, c** TEM images of SP-C. The SP-C sample was prepared by etching Fe_3O_4 from SP-Fe_3O_4-C; the etching steps referred to the reported literature.[12] ICP-MS-determined Fe content in SP-C was only 0.12 wt%. The XRD curve of SP-C shows two broad peaks of carbon while no peaks of Fe_3O_4. In addition, there was no Fe_3O_4 particle in the TEM images of SP-C. These results indicate that the Fe_3O_4 particles have been removed.

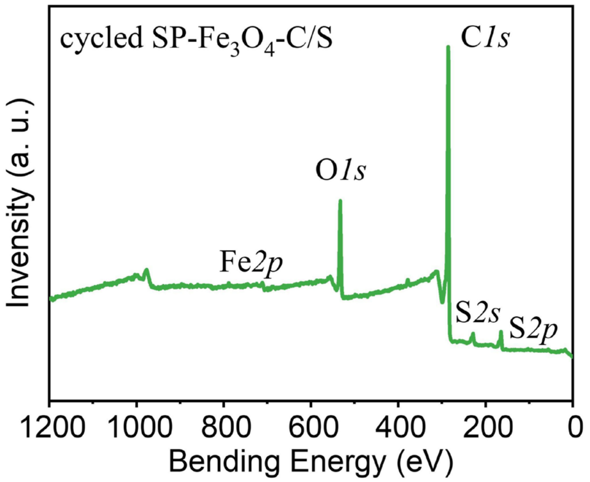

Supplementary Fig. 19. XPS survey spectrum of the cycled SP-Fe_3O_4-C/S cathode.

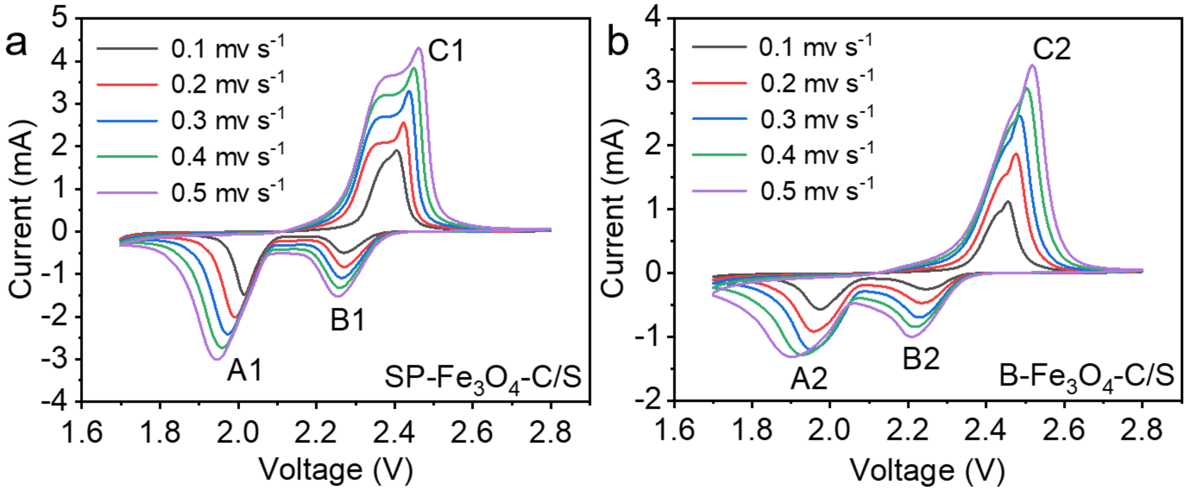

Supplementary Fig. 20. Representative voltammograms at different scan rates: a SP-Fe_3O_4-C/S. **b** B-Fe_3O_4-C/S.

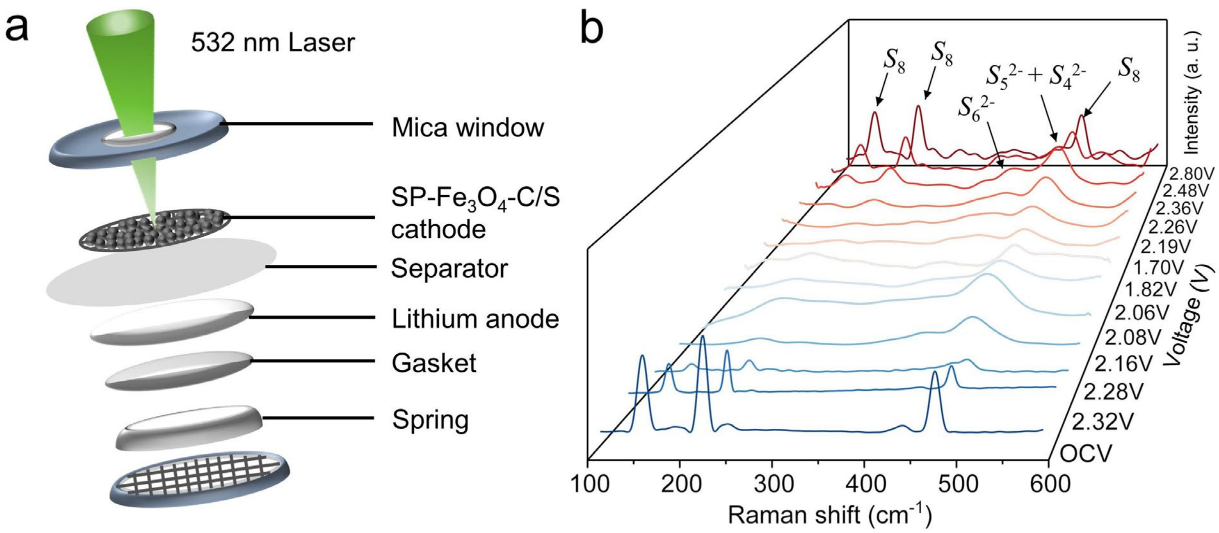

Supplementary Fig. 21. *In situ* Raman system: a Cell configuration for *in situ* Raman observation. **b** *In situ* Raman spectra at 0.1 C and different voltages for the SP-Fe_3O_4-C/S-based battery.

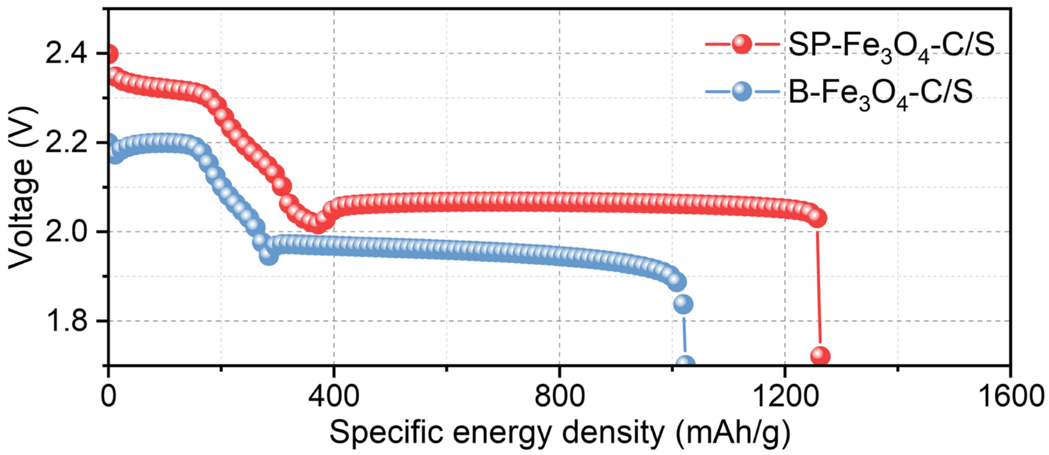

Supplementary Fig. 22. Simulated discharge curves of the SP-Fe_3O_4-C/S and B-Fe_3O_4-C/S models.

Supplementary Tables

Supplementary Table. 1. Structural parameters of SP-Fe_3O_4-C, SP-Fe_3O_4-C/S, B-Fe_3O_4-C, and B-Fe_3O_4-C/S obtained from the nitrogen adsorption-desorption isotherms

Samples	S_{BET} [m^2 g^{-1}][a)]	S_{micro} [m^2 g^{-1}][b)]	$S_{external}$ [m^2 g^{-1}][c)]	V_{total} [cm^3 g^{-1}][d)]	V_{micro} [cm^3 g^{-1}][e)]	$V_{external}$ [cm^3 g^{-1}][f)]	D_{micro} [nm][g)]	D_{meso} [nm][h)]
SP-Fe_3O_4-C	485	144	341	1.08	0.11	0.97	0.3	30
SP-Fe_3O_4-C/S	49	0.02	48.98	0.31	0	0.31	0	20
B-Fe_3O_4-C	418	299	119	0.64	0.10	0.54	0.8	17
B-Fe_3O_4-C/S	1	0	1	0.0032	0	0.0032	0	30

[a)]Specific surface area calculated by the BET method. [b)]Specific surface area of the micropores calculated by the *V*-*t* plot method. [c)]External surface area. [d)]Total pore volume. [e)]Micropore volume calculated by the *V*-*t* plot method. [f)]External pore volume. [g)]The average diameter of micropores is calculated by the nonlocal DFT method from the adsorption branch of the nitrogen adsorption-desorption isotherm. [h)]The average diameter of mesopores is calculated by the BJH method based on the adsorption branch of the nitrogen adsorption-desorption isotherm.

Supplementary Table. 2. Comparison of electrochemical performance of the Li-S battery in this study with those in the previously reported Li-S batteries based on PCP-based sulfur cathodes

Host	Capacity (Low rate) [mAh g^{-1}]	Capacity (High rate) [mAh g^{-1}]	Cycle number	Decay Rate Per cycle	Capacity retention	Sulfur loading [mg cm^{-2}]	Areal sulfur capacity [mAh cm^{-2}]	Reference
SP-Fe_3O_4-C	1303.4 (0.2 C)	691.8 (5 C)	1200 (1 C)	0.027%	67.6%	8.2	6.5 (0.1 C)	This work
α-Ta_2O_{5-x}/MCN	1200.0 (0.2 C)	766.0 (5 C)	1000 (1 C)	0.029%	71%	5.6	5.0 (0.2 C)	13
Fe_3O_4@CNT nano spheres	942.2 (0.2 C)	667.6 (2 C)	1800 (1 C)	0.023%	58%	5.5	4.1 (0.2 C)	14
PCMS	941.0 (0.2 C)	480.0 (4 C)	900 (2 C)	0.060%	46%	4.3	4.0 (0.2 C)	15
HMCS/S@GO	976.0 (0.2 C)	626.0 (2 C)	300 (1 C)	0.203%	61%	N/A	N/A	16
N-PC@uCo	1180.0 (0.2 C)	600.0 (5 C)	500 (1 C)	0.028%	86%	5.9	4.8 (0.2 C)	17
HDS-HSs Mo_2C/C	1174.3 (0.2 C)	641.9 (5 C)	500 (1 C)	0.016%	92%	5.8	5.9 (0.2 C)	18
yolk-shell ZnSe-$CoSe_2$	1127.8 (0.2 C)	403.6 (5 C)	400 (1 C)	0.126%	50.6%	6.1	4.2 (0.2 C)	19
HCMS	1162.0 (0.2 C)	360.0 (5 C)	900 (1 C)	0.040%	57.3%	4.8	4.0 (0.1 C)	20
Ni-NC(p)	924.7 (0.2 C)	706.2 (2 C)	600 (0.5 C)	0.078%	46.8%	N/A	N/A	21
CoSAs@NC	1239.0 (0.2 C)	764.0 (5 C)	600 (1 C)	0.046%	72.5%	5.0	2.0 (1 C)	22
ZnS/SnS_2@NC	1283.0 (0.2 C)	607.0 (5 C)	500 (1 C)	0.016%	92.6%	5.9	3.8 (0.5 C)	23
Ti_4O_7/TiN/C	1204.5 (0.2 C)	616.5 (4 C)	1000 (4 C)	0.033%	66.6%	N/A	N/A	24
Fe-CoNC	1427.0 (0.2 C)	618.0 (10 C)	2800 (2 C)	0.01%	72.0%	8.0	4.9 (0.2 C)	25

Fe_xN@C	1148.7 (0.2 C)	858.1 (2 C)	500 (1 C)	0.095%	76.7%	N/A	N/A	26
TI-NHCNS	1171.0 (0.2 C)	610.0 (5 C)	600 (1 C)	0.053%	68.2%	N/A	N/A	4
Fe_3C/OMMCNS	1095 (0.2 C)	656 (5 C)	1000 (1 C)	0.033%	67.0%	4.0	3.3 (0.2 C)	27
Fe-N/MHCS	1110.0 (0.2 C)	949.0 (2 C)	1000 (1 C)	0.019%	82.9%	5.4	5.2 (0.1 C)	28
$Fe_{1-x}S$-NC	1106.0 (0.5 C)	628.0 (5 C)	200 (0.5 C)	0.003%	99.4%	8.1	5.1 (0.05 C)	29
MHCS	1139.0 (0.2 C)	476.0 (2 C)	3100 (0.5 C)	0.023%	28.7%	4.1	N/A	30
NHCS-SnS_2	1047.3 (0.2 C)	515.6 (2 C)	500 (1 C)	0.037%	81.3%	N/A	N/A	31
FeCo DACs	1233.0 (0.2 C)	688.0 (5 C)	1000 (1 C)	0.018%	82%	8.7	9.6 (0.1 C)	32
TiO_2-TiN/C	1171.0 (0.2 C)	690.0 (4 C)	800 (2 C)	0.025%	80%	8.0	5.12 (0.2 C)	33
CoS_2@NGCNs	1003.8 (0.2 C)	525.3 (2 C)	300 (1 C)	0.075%	77%	N/A	N/A	34
HNPC-900-65s	1173.0 (0.2 C)	623.0 (5 C)	800 (2 C)	0.035%	72%	N/A	N/A	35
Co/Co_3O_4-NHC	957.1 (0.2 C)	447.9 (5 C)	500 (1 C)	0.032%	84.1%	4.0	1.5 (1 C)	36
A-Nb_2O_{5-x}@MCS	1210.3 (0.2 C)	941.9 (5 C)	1200 (1 C)	0.024%	71.2%	5.8	6.6 (0.1 C)	37
$Fe_{3-x}C$@C	1265.0 (0.2 C)	609.0 (5 C)	1000 (1 C)	0.040%	60.0%	7.0	5.6 (0.1 C)	38
Co-P cluster/NC	1216.0 (0.2 C)	623.0 (6 C)	1000 (1 C)	0.022%	78.0%	6.2	6.5 (0.1 C)	39
3d-omsh/ZnS, Co-N-C	~1300.0 (0.2 C)	~650.0 (5 C)	1000 (1.6 C)	0.022%	~77.0%	9.0	6.5 (0.1 C)	40
Co@N-HCMSs	1089.0 (0.2 C)	692.0 (4 C)	500 (1 C)	0.080%	60%	5.1	5.0 (0.1 C)	41

FPHCs	958.0 (0.2 C)	720.0 (2 C)	500 (2 C)	0.054%	73%	3.5	1.9 (2C)	42
Mo_2C-C NOs	1219.0 (0.2 C)	337.0 (5 C)	600 (1 C)	0.046%	73%	4.2	2.6 (0.5 C)	43
N-Co_3O_4@N-C/GO	1301.0 (0.1 C)	652.0 (3 C)	1000 (2 C)	0.020%	80%	5.9	3.3 (0.2 C)	44

Supplementary Table. 3. Comparison of electrochemical performance of our work with previously reported Li-S batteries focused on carbon-based sulfur cathode development

Host	Capacity (Low rate) mAh g^{-1}	Capacity (High rate) mAh g^{-1}	Cycle number	Decay Rate Per cycle	Capacity retention	Sulfur loading (mg cm^{-2})	Areal sulfur capacity (mAh cm^{-2})	Reference
SP-Fe_3O_4-C	1303.4 (0.2 C)	691.8 (5 C)	1200 (1 C)	0.027%	67.6%	8.2	6.5 (0.1 C)	This work
$NiMoO_4$@NSCC	1336.4 (0.1 C)	691.1 (1 C)	500 (1 C)	0.008%	96.0%	5.0	2.80 (1 C)	45
CoFe-MCS	1099.2 (0.2 C)	671.4 (2 C)	500 (2 C)	0.062%	69.0%	7.7	6.0 (0.1 C)	46
ALDVO@3DNG	1555.0 (0.2 C)	195.0 (5 C)	350 (2 C)	0.052%	81.8%	11.5	14.9 (0.2 C)	47
Fe_2O_3/N-MC	1172.0 (0.2 C)	740.0 (5 C)	1000 (5 C)	0.013%	87.0%	6.5	5.35 (1 C)	48
CoSe@BNCNTs/CC	1326.0 (0.2 C)	683.0 (6 C)	2000 (1 C)	0.011%	78.0%	7.9	9.76 (0.1 C)	49
MOF-Co_4N	1425.0 (0.1 C)	729.0 (3 C)	400 (1 C)	0.044%	82.4%	N/A	N/A	50
NbC/CoCN-PCFs	1210.0 (0.2 C)	640.0 (5 C)	500 (2 C)	0.063%	68.5%	6.7	6.1 (0.2 C)	51
CoSe@C	860.0 (0.2 C)	715 (1 C)	600 (1 C)	0.040%	76.0%	6.2	5.8 (0.2 C)	52
A-3DNG	1101.0 (0.2 C)	1000 (2 C)	200 (1 C)	0.053%	89.4%	N/A	N/A	53
RF-TiN	1338.0	690	800	0.040%	68.0%	7.0	5.9	54

	(0.2 C)	(5 C)	(1 C)				(0.2 C)	
NHSC	1047.3 (0.2 C)	515.6 (2 C)	500 (1 C)	0.037%	81.5%	N/A	N/A	55
RHCF/CoO	1121.0 (0.2 C)	663.4 (5 C)	500 (1 C)	0.048%	76.1%	N/A	N/A	55
TiN-VN@CNFs	1385.0 (0.2 C)	650.0 (5 C)	600 (2 C)	0.051%	69.4%	5.6	5.5 (0.1 C)	56
MCG	1103.0 (0.2 C)	763.0 (2 C)	710 (1 C)	0.056%	60.2%	3.5	4.0 (0.2 C)	57
N/O doped HCMs	1162.0 (0.2 C)	227.0 (10 C)	900 (1 C)	0.040%	64.0%	4.8	4.0 (0.1 C)	20
Fe_3C/NC	1176.0 (0.2 C)	825.0 (2 C)	100 (0.5 C)	0.141%	85.9%	5.0	3.6 (0.2 C)	58
CPC@FeS_2	1459.0 (0.1 C)	916.0 (2 C)	900 (1.5 C)	0.043%	61.3%	8.4	8.5 (0.1 C)	59
CMCS	1260.0 (0.2 C)	485.0 (3 C)	200 (2 C)	0.013%	97.4%	N/A	N/A	60
MCCN@C	1163.0 (0.2 C)	626.0 (5 C)	1000 (2 C)	0.032%	68.0%	3.6	3.4 (0.2 C)	61
Ni-NC(p)	924.7 (0.2 C)	706.2 (2 C)	600 (0.5 C)	0.078%	53.2%	N/A	N/A	21
Z-CoS_2	910.0 (0.2 C)	430.0 (5 C)	1000 (1 C)	0.040%	60.0%	2.9	3.0 (0.2 C)	62
C@PtNi 2h	1093.8 (0.2 C)	545.7 (2 C)	300 (1 C)	N/A	N/A	2.8	1.6 (0.2 C)	63
VC/V_2O_{3-X}	1161.0 (0.2 C)	882.0 (5 C)	1000 (1 C)	0.020%	80.0%	7.0	6.29 (0.2 C)	64
HMCS@GO	976.0 (0.2 C)	626.0 (2 C)	300 (0.2 C)	0.160%	52.0%	N/A	N/A	16
Co single atom @ NC	1239.0 (0.2 C)	670.0 (10 C)	600 (1 C)	0.046%	72.4%	N/A	5.0	22
Co-NPC-MCs	1120.0 (0.2 C)	836.0 (2 C)	400 (1 C)	0.070%	72.0%	4.9	3.5 (0.5 C)	11
Fe SAs @ BCN	1356.0 (0.2 C)	690.0 (5 C)	1000 (5 C)	0.018%	82.0%	12.0	11.3 (0.2 C)	65

N/O-HC900	1241.0 (0.2 C)	958.0 (3 C)	500 (1 C)	0.110%	45.0%	7.4	7.35 (0.2 C)	66
C_{FS}	1428.0 (200 mA g^{-1})	380.0 (5 A/g)	500 (500 mA g^{-1})	0.036%	82.0%	4.0	6.7 (100 mA g^{-1})	67
Ti_4O_7/TiN/C	1204.5 (0.2 C)	616.5 (4 C)	1000 (1 C)	0.014%	86.0%	3.0	N/A	24
Ni_{SA}-Ni_{NP}-CDs@CNF	1150.0 (0.2 C)	851.0 (2 C)	500 (1 C)	0.038%	81.0%	50.0	66.5 (0.02 C)	68
Hollow carbon nanofibers	1264.0 (0.5 C)	860.0 (4 C)	500 (2 C)	0.078%	61.0%	N/A	N/A	69
3DP CNTs-CO_2	685.0 (0.2 C)	252.1 (2 C)	400 (0.1 C)	0.123%	50.8%	10.0	5.74 (0.1 C)	70
Co_4N/WCP	1280.5 (0.2 C)	841.7 (2 C)	500 (1 C)	0.029%	85.5%	4.0	5.0 (5 mA cm^{-2})	71
MnO@PNC	1138.0 (0.2 C)	802.0 (5 C)	520 (1 C)	0.034%	82.3%	3.0	N/A	72
Co/N-PCNSs	1234.0 (0.2 C)	411.0 (5 C)	400 (5 C)	0.036%	85.6%	N/A	N/A	73
ZnS/SnS_2@NC	1294.0 (0.2 C)	607.0 (5 C)	500 (1 C)	0.016%	92.0%	5.9	4.77 (0.5 C)	23
C-Co/TiO_2	638.6 (0.5 C)	383.8 (3 C)	300 (1 C)	0.051%	84.7%	1.5	0.96 (0.5 C)	74
Fe_xN@C	1148.7 (0.2 C)	858.1 (2 C)	500 (1 C)	0.095%	52.5%	N/A	N/A	26
NPC/G hybrid	932 (0.2 C)	786.0 (1 C)	300 (1 C)	0.043%	87.1%	2.4	N/A	75
NCCNT-Co	1103.0 (0.2 C)	845.0 (2 C)	500 (1 C)	0.024%	88.0%	N/A	N/A	76
Cu_xS@NC/S-F	1266.0 (0.2 C)	729.0 (4 C)	500 (2 C)	0.058%	71.0%	3.5	3.83 (1 C)	10
Mo-N-CNF	1248.0 (0.2 C)	715.0 (5 C)	400 (1 C)	0.062%	75.2%	5.1	6.24 (0.1 C)	77

SACNTs@SNC	1175.0 (0.2 C)	800.0 (5 C)	1500 (2 C)	0.037%	44.5%	7.0	6.3 (0.5 C)	78
Ni_3S_2/(N,S)-RGD	1335.4 (0.2 C)	826.2 (5 C)	1000 (3 C)	0.023%	77.0%	5.8	6.72 (0.05 C)	79
rod-TiO_2@C	1017.0 (0.2 C)	509.0 (10 C)	1500 (1 C)	0.020%	70.0%	4.04	3.72 (0.5 C)	80
CN@NSHPC	1447.0 (0.2 C)	387.0 (5 C)	500 (1 C)	0.048%	76.0%	2.0	2.89 (0.2 C)	81
CPC@FeS_2	1459.0 (0.1 C)	916.0 (2 C)	900 (1.5 C)	0.043%	61.3%	8.4	8.5 (0.1 C)	59
MCCN@C	1234.0 (0.2 C)	626.0 (5 C)	1000 (2 C)	0.032%	68.0%	3.6	3.39 (0.2 C)	61
M-GNTs	909.0 (0.2 C)	668.9 (2 C)	500 (1 C)	0.080%	60.0%	4.5	4.07 (0.2 C)	82
HMCS@GO	976.0 (0.2 C)	626.0 (2 C)	300 (0.2 C)	0.160%	52.0%	N/A	N/A	16
Co/N-PCNSs	871.0 (0.5 C)	520.0 (5 C)	400 (5 C)	0.036%	85.6%	N/A	N/A	73
VO_2/V_3S_4@LPC	1111.0 (0.2 C)	525.0 (3 C)	1000 (1 C)	0.016%	84.0%	4.2	3.8 (0.2 C)	83
Co_9S_8@C NBs	1127.0 (0.2 C)	802.0 (2 C)	400 (1 C)	0.043%	82.8%	1.6	N/A	84
DHPCs	1005.0 (0.2 C)	746.0 (2 C)	500 (2 C)	0.060%	70.0%	N/A	N/A	85
H-MoC-NC	1025.0 (0.2 C)	646.0 (3 C)	1000 (3 C)	0.049%	51.0%	3.01	3.33 (0.1 C)	86
Fe_3C@C@Fe_3C	1490.0 (0.2 C)	750.0 (2 C)	1000 (0.5 C)	0.050%	50.0%	N/A	N/A	87
TI-NHCNS	1200.0 (0.2 C)	540.0 (5 C)	1000 (1 C)	0.044%	56.0%	4.0	4.4 (0.05 C)	4
Fe-N_5-C	1170.0 (0.2 C)	723.0 (3 C)	500 (1 C)	0.054%	73.0%	8.2	6.0 (0.1 C)	5
Fe_2N@C NBs	1361.0 (0.1 C)	778.0 (3 C)	600 (1 C)	0.036%	78.4%	4.1	4.2 (0.5 C)	6

Fe-N-C-MCF	892.0 (0.2 C)	504.0 (5 C)	500 (3 C)	0.038%	81.0%	5.2	2.5 (3 C)	7
ePCNTM	1178.0 (0.2 C)	785.0 (3 C)	500 (1 C)	0.046%	77.0%	6.5	3.2 (3 C)	8
Mo_2C/CHS	1441.0 (0.1 C)	904.0 (1.5 C)	200 (0.5 C)	0.055%	89.0%	5.0	4.0 (0.5 C)	88
IRA-DC	1225.5 (0.5 C)	473.6 (20 C)	1200 (1 C)	0.049%	41.2%	13.1	8.86 (0.5 mA cm^{-2})	89
yolk-shell ZnSe-$CoSe_2$	1127.8 (0.2 C)	403.6 (5 C)	400 (1 C)	0.126%	50.6%	6.08	4.16 (0.2 C)	9
Fe-CoNC	1427.0 (0.2 C)	618.0 (10 C)	2800 (2 C)	0.01%	72.0%	8.0	4.9 (0.2 C)	25

Supplementary Table. 4. The Li-ion diffusion coefficients with different sulfur hosts.

Li-Ion Diffusion Coefficient (10×10^{-9} cm^2 s^{-1})	1: SP- Fe_3O_4-C	2: B- Fe_3O_4-C
Peak A	18.3	5.39
Peak B	8.12	5.10
Peak C	47.4	41.8

Calculations and Simulations

Density Function Theory calculation

The first-principles calculations were conducted by using Vienna Ab-initio Simulation Package (VASP) software with density functional theory (DFT) and projector augmented-wave plane-wave (PAW) pseudopotential method.[90] The generalized gradient approximation (GGA) with the Perdew–Burke–Ernzerhof (PBE) function was applied to describe the electron exchange-correlation functions.[91] The surface of Fe_3O_4 (220) had a vacuum thickness of 15 Å in the z-direction to avoid interaction between the slabs. The kinetic energy cutoff of electron wave

functions was used as 400 eV. The K-point meshes used for the first Brillouin zone integration were generated by Monkhorst-Pack scheme as 2 × 3 × 1. The model structure with the fixed supercell was optimized using thresholds for the total energy of 10^{-4} eV and the convergence criterion of atomic forces was set to 0.03 eV $Å^{-1}$. DFT-D3 of dispersion correction was adopted to describe the van der Waals (*vdW*) interactions.

The adsorption energy (E_{ads}) was used to measure the strength of the interaction between the surface of catalyst and sulfur species, and is calculated using the following formula:

$$E_{ads} = E_{(substrate\ and\ LiPSs)} - E_{(substrate)} - E_{(LiPSs)} \quad (1)$$

In the formula, $E_{(substrate\ and\ LiPSs)}$ is the total energy of the adsorption configuration between the catalyst surface and polysulfide; $E_{(substrate)}$ is the energy of the isolated catalyst surface; $E_{(LiPSs)}$ is the energy of the isolated polysulfide.

Finite element simulation

The simulation process of Li-S battery model is performed by finite element method based on COMSOL Multiphysics 6.0 platform.[92] The following is the main equations used in the simulation.

Mass transport

For the transport of different ions in the liquid phase, it is driven by the migration because of electric field and the diffusion because of concentration gradient, which can be expressed by the Nernst−Planck equation:

$$N_{i,m} = -D_{i,m}(\nabla C_i^b + \frac{z_i F C_i^b}{RT} \nabla \phi_e) \quad (2)$$

where $D_{i,m}$ is the diffusion coefficient of species i ($i = Li^+$, $S_{8(l)}$, $S_8{}^{2-}$, $S_6{}^{2-}$, $S_4{}^{2-}$, $S_2{}^{2-}$, S^{2-}, and A^-, which is the anion of the lithium salt used in the electrolyte) and the index m corresponds to each domain (*sep* for separator and *cat* for cathode). $N_{i,m}$ is ions flux, C_i^b is the concentration of species i in the electrolyte, z_i the charge number of species i, and ϕ_e is the electrolyte potential.

Charge transfer

At the interface of the electrolyte and the anode, the reaction process of Li-ions can be described by the simplified reaction:

$$Li^{+} + e^{-} \rightleftharpoons Li \tag{3}$$

On the cathode side, the solid sulfur ($S_{8(s)}$) in the active particles dissolves into the electrolyte and forms $S_{8(l)}$, which would be reduced to lower order polysulfide anions through a chain of electrochemical reactions as follows:

$$S_{8(s)} \rightleftharpoons S_{8(l)} \tag{4}$$

$$\frac{1}{2}S_{8(l)} + e^{-} \rightleftharpoons \frac{1}{2}S_{8}^{2-} \tag{5}$$

$$\frac{3}{2}S_{8}^{2-} + e^{-} \rightleftharpoons 2S_{6}^{2-} \tag{6}$$

$$S_{6}^{2-} + e^{-} \rightleftharpoons \frac{3}{2}S_{4}^{2-} \tag{7}$$

$$\frac{1}{2}S_{4}^{2-} + e^{-} \rightleftharpoons S_{2}^{2-} \tag{8}$$

$$\frac{1}{2}S_{2}^{2-} + e^{-} \rightleftharpoons S_{\square}^{2-} \tag{9}$$

The charge transfer rate of the electrochemical reaction can be indicated by the local current density, for each reaction j, which is given by the Butler-Volmer equation:

$$i = i_{ex,j}[\prod\left(\frac{C_i}{C_{i,ref}}\right)^{pi,j} \exp\left(\frac{\alpha_{a,j}F\eta_j}{RT}\right) - \prod\left(\frac{C_i}{C_{i,ref}}\right)^{qi,j} \exp\left(\frac{\alpha_{c,j}F\eta_j}{RT}\right)] \tag{10}$$

where $i_{ex,j}$ is exchange current density, $\alpha_{a,j}$ and $\alpha_{c,j}$ are the anodic and cathodic charge transfer coefficients, respectively, $C_{i,ref}$ is the reference concentration and C_i is the concentration of each specie i near the interface between electrode and electrolyte, η_j is overpotential, which can be calculated from:

$$\eta = \phi_s - \phi_e - U_{eq} \tag{11}$$

Where ϕ_s and ϕ_s is the solid phase potential and electrolyte potential, U_{eq} is the equilibrium potential of the battery.

Thermodynamics

The open-circuit potential for reaction j at the reference concentrations $C_{i,ref}$ assumed to be equal to initial concentration of species i is given by the Nernst equation:

$$U_{j,ref} = U_j^{\theta} - \frac{RT}{n_jF}\sum_i\left\{s_{i,j}ln\left[\frac{C_{i,ref}}{1000}\right]\right\} \tag{12}$$

U_j^{θ} is the standard equilibrium potential of each reaction.

Precipitation/Dissolution of S_8 and Li_2S

The rate of precipitation of species Li_2S and S_8, assuming that the precipitation reaction is kinetically controlled can be written as:

$$R_k = K_k(\prod_i C_i^{\gamma_{i,k}} - K_{sp,k}) \tag{13}$$

Where R_k is the rate of precipitation of the solid species (k=Li_2S, S_8), $K_{sp,k}$ is the solubility product of k in the electrolyte and K_k is the rate constant and $\gamma_{i,k}$ is the number of moles of ionic species i in the solid species k.

Mechanical behavior

In the mechanical simulation, fixed constraints ($u = 0$) are set at both ends of the battery to simulate the role of the fixture, the other components on the battery surface are set as roller constraint ($u * \boldsymbol{n} = 0$), and the cathode surface is given a displacement in the normal direction according to the precipitation of Li_2S which can be calculated according to the velocity u_n:

$$u_n = AR_{Li_2S}\frac{M}{\rho} \tag{14}$$

where M and ρ is the molar mass and density of Li_2S, A is the surface area. The kinematic stress strain constitutive relation is given by Hooke's Law:

$$\sigma_{ij} = \frac{E}{1+\upsilon}\nabla\vec{\ell} + \frac{2\upsilon E}{1-2\upsilon} \tag{15}$$

where E is Young's modulus, $\nabla\vec{\ell}$ is the displacement of the Cathode-electrolyte interface and υ is Poisson's ratio.

The simulation parameters could be seen at the **Supplementary Table. 5-8**. For the cathode utilization calculation, it is defined as the ratio of volume fraction of Li_2S actually generated compared to the volume fraction of lithium disulfide generated theoretically. The radius of cathode S/C particles is set as 1um, and the radius of 3D channels is set as 0.1um.

Supplementary Table. 5. Kinetic and thermodynamic properties in the FEM simulation

Reaction [j]	$i_{0j,ref}$ [A m^{-2}]	$a_{a,j}$	$a_{c,j}$	n_j	U_j [V]
**(1)**	0.394	0.5	0.5	1	0.0
**(2)**	1.972	0.5	0.5	1	2.39
**(3)**	0.019	0.5	0.5	1	2.37
**(4)**	0.019	0.5	0.5	1	2.24
**(5)**	1.97E-4	0.5	0.5	1	2.04
**(6)**	1.97E-7	0.5	0.5	1	2.01

Supplementary Table. 6. Transport properties and reference concentrations in the FEM simulation

Specie [i]	z_i	$D_{i,0}$ [m^{2}s^{-1}]	$C_{i,ref}$ [mol m^{-3}]
Li^+	1	1E-10	1001.04
$S_{8(l)}$	0	1E-9	19
S_8^{2-}	-2	6E-10	0.178
S_6^{2-}	-2	6E-10	0.324
S_4^{2-}	-2	1E-10	0.02
S_2^{2-}	-2	1E-10	5.229E-7
S^{2-}	-2	1E-10	8.267E-10
A^-	-1	4E-10	1000

Supplementary Table. 7. Parameters for precipitation reactions in the FEM simulation

Precipitate	Rate Constant	Solubility Product	Molar Volume
$S_{8(S)}$	1[s^{-1}]	19 [mol m^{-3}]	1.239E-4
$Li_2S_{(s)}$	1E-4[m^6 mol^2 s^{-1}]	1E2[mol^3 m^{-9}]	2.768E-5

Supplementary Table. 8. Mechanical parameters in the FEM simulation

Subject	Parameter	Value	Unit
**Separator**	Young's modulus	1	GPa
	Poisson's ratio	0.25	1
	Density	1011	kg/m^3
**Li metal**	Young's modulus	2	GPa
	Poisson's ratio	0.34	1
	Density	534	kg/m^3
	Molar mass	0.030	kg/mol
**Cathode**	Young's modulus	5	GPa
	Poisson's ratio	0.25	1
	Density	2360	kg/m^3
**Conductive materials**	Young's modulus	0.84	GPa
	Poisson's ratio	0.38	1
	Density	25	kg/m^3
**S_8 molecule**	Density	2069	kg/m^3
	Molar mass	0.046	kg/mol
**Li_2S molecule**	Density	1915	kg/m^3

Reference

1. Xiang, L. et al. Porous polymer cubosomes with ordered single primitive bicontinuous architecture and their sodium–iodine batteries. *J. Am. Chem. Soc.* **144**, 15497-15508 (2022).
2. Zhang, W.-M. et al. Carbon coated Fe_3O_4 nanospindles as a superior anode material for lithium-ion batteries. *Adv. Funct. Mater.* **18**, 3941-3946 (2008).
3. Gao, G. et al. One-pot synthesis of carbon coated Fe_3O_4 nanosheets with superior lithium storage capability. *J. Mater. Chem. A* **3**, 4716-4721 (2015).
4. Yu, Z. et al. Radially inwardly aligned hierarchical porous carbon for ultra-long-life lithium–sulfur batteries. *Angew. Chem., Int. Ed.* **59**, 6406-6411 (2020).
5. Zhang, Y. et al. Engineering oversaturated Fe-N_5 multifunctional catalytic sites for durable lithium-sulfur batteries. *Angew. Chem., Int. Ed.* **60**, 26622-26629 (2021).
6. Sun, W. et al. Rational construction of Fe_2N@C yolk–shell nanoboxes as multifunctional hosts for ultralong lithium–sulfur batteries. *ACS Nano* **13**, 12137-12147 (2019).
7. Lim, W.-G. et al. Synergistic effect of molecular-type electrocatalysts with ultrahigh pore volume carbon microspheres for lithium–sulfur batteries. *ACS Nano* **12**, 6013-6022 (2018).
8. Zhang, Y. et al. "Sauna" activation toward intrinsic lattice deficiency in carbon nanotube microspheres for high-energy and long-lasting lithium–sulfur batteries. *Adv. Energy Mater.* **11**, 2100497 (2021).
9. Xu, J. et al. Heterostructure ZnSe-$CoSe_2$ embedded with yolk-shell conductive dodecahedral as two-in-one hosts for cathode and anode protection of lithium–sulfur full batteries. *Energy Stor. Mater.* **47**, 223-234 (2022).
10. Yu, Q. et al. In situ formation of copper-based hosts embedded within 3D N-doped hierarchically porous carbon networks for ultralong cycle lithium–sulfur batteries. *Adv. Funct. Mater.* **28**, 1804520 (2018).
11. Wang, P. et al. Pomegranate-like microclusters organized by ultrafine Co nanoparticles@nitrogen-doped carbon subunits as sulfur hosts for long-life lithium–sulfur batteries. *J. Mater. Chem. A* **6**, 14178-14187 (2018).
12. He, J., Luo, L., Chen, Y., Manthiram, A. Yolk–shelled C@Fe_3O_4 nanoboxes as efficient sulfur hosts for high-performance lithium–sulfur batteries. *Adv. Mater.* **29**, 1702707 (2017).
13. Zhang, Z. et al. Tantalum-based electrocatalyst for polysulfide catalysis and retention for high-performance lithium-sulfur batteries. *Matter* **3**, 920-934 (2020).
14. Zhang, Y. et al. Long-life Li–S batteries based on enabling the immobilization and catalytic conversion of polysulfides. *J. Mater. Chem. A* **7**, 21747-21758 (2019).
15. Liu, S. et al. 3D pomegranate-like structures of porous carbon microspheres self-assembled by hollow thin-walled highly-graphitized nanoballs as sulfur immobilizers for Li–S batteries. *Nano Energy* **63**, 103894 (2019).
16. Zhe, R. et al. Graphene oxide wrapped hollow mesoporous carbon spheres as a dynamically bipolar host for lithium–sulfur batteries. *J. Mater. Chem. A* **10**, 24422-24433 (2022).
17. Wang, R. et al. Highly dispersed cobalt clusters in nitrogen-doped porous carbon enable multiple effects for high-performance Li–S battery. *Adv. Energy Mater.* **10**, 1903550 (2020).

18. Li, W. et al. Mo_2C/C hierarchical double-shelled hollow spheres as sulfur host for advanced li-s batteries. *Angew. Chem., Int. Ed.* **60**, 21512-21520 (2021).
19. Feng, L. et al. Regulating polysulfide diffusion and deposition via rational design of core–shell active materials in Li–S batteries. *ACS Nano* **16**, 7982-7992 (2022).
20. Zhou, X. et al. N/O dual-doped hollow carbon microspheres constructed by holey nanosheet shells as large-grain cathode host for high loading Li-S batteries. *Energy Stor. Mater.* **24**, 644-654 (2020).
21. Li, Y. et al. Mesoporous N-rich carbon with single-Ni atoms as a multifunctional sulfur host for Li-S batteries. *Angew. Chem., Int. Ed.* **61**, e202212680 (2022).
22. Li, Y. et al. Cobalt single atoms supported on N-doped carbon as an active and resilient sulfur host for lithium–sulfur batteries. *Energy Stor. Mater.* **28**, 196-204 (2020).
23. Yan, B. et al. Confining ZnS/SnS_2 ultrathin heterostructured nanosheets in hollow N-doped carbon nanocubes as novel sulfur host for advanced Li-S batteries. *Small* **18**, 2107727 (2022).
24. Ma, L. et al. Construction of Ti_4O_7/TiN/carbon microdisk sulfur host with strong polar N–Ti–O bond for ultralong life lithium–sulfur battery. *Energy Stor. Mater.* **44**, 180-189 (2022).
25. Dong, C. et al. Boosting Bi-directional redox of sulfur with dual metal single atom pairs in carbon spheres toward high-rate and long-cycling lithium–sulfur battery. *Adv. Energy Mater.* **13**, 2301505 (2023).
26. Xie, D. et al. Poly(ionic liquid) nanovesicle-templated carbon nanocapsules functionalized with uniform iron nitride nanoparticles as catalytic sulfur host for Li–S batteries. *ACS Nano* **16**, 10554-10565 (2022).
27. Jin, Z. et al. Ordered micro-mesoporous carbon spheres embedded with well-dispersed ultrafine Fe_3C nanocrystals as cathode material for high-performance lithium-sulfur batteries. *Chem. Eng. J.* **388**, 124315 (2020).
28. Shao, Q. et al. Atomic level design of single iron atom embedded mesoporous hollow carbon spheres as multi-effect nanoreactors for advanced lithium–sulfur batteries. *J. Mater. Chem. A* **8**, 23772-23783 (2020).
29. Boyjoo, Y. et al. Molecular-level design of pyrrhotite electrocatalyst decorated hierarchical porous carbon spheres as nanoreactors for lithium–sulfur batteries. *Adv. Energy Mater.* **10**, 2000651 (2020).
30. Hou, J. et al. Remarkable cycling durability of lithium-sulfur batteries with interconnected mesoporous hollow carbon nanospheres as high sulfur content host. *Chem. Eng. J.* **401**, 126141 (2020).
31. Yang, J.-L. et al. Catalytic interfaces-enriched hybrid hollow spheres sulfur host for advanced Li–S batteries. *Adv. Mater. Interfaces* **7**, 1901420 (2020).
32. Fu, W. et al. Photoinduced loading of electron-rich Cu single atoms by moderate coordination for hydrogen evolution. *Nat. Commun.* **13**, 5496 (2022).
33. Xu, Z.-L. et al. Visualization of regulated nucleation and growth of lithium sulfides for high energy lithium sulfur batteries. *Energy Environ. Sci.* **12**, 3144-3155 (2019).
34. Seo, S.-D., Park, D., Park, S., Kim, D.-W. "Brain-coral-like" mesoporous hollow CoS_2@N-doped graphitic carbon nanoshells as efficient sulfur reservoirs for lithium–sulfur batteries. *Adv.*

Funct. Mater. **29**, 1903712 (2019).
35. Li, Z. et al. Engineered interfusion of hollow nitrogen-doped carbon nanospheres for improving electrochemical behavior and energy density of lithium–sulfur batteries. *Adv. Funct. Mater.* **29**, 1902322 (2019).
36. Jeon, Y. et al. Co/Co_3O_4-embedded N-doped hollow carbon composite derived from a bimetallic MOF/ZnO core-shell template as a sulfur host for Li-S batteries. *Chem. Eng. J.* **407**, 126967 (2021).
37. Luo, D. et al. Revealing the rapid electrocatalytic behavior of ultrafine amorphous defective Nb_2O_{5-x} nanocluster toward superior Li–S performance. *ACS Nano* **14**, 4849-4860 (2020).
38. Zhang, Y. et al. Hierarchical defective $Fe_{3-x}C$@C hollow microsphere enables fast and long-lasting lithium–sulfur batteries. *Adv. Funct. Mater.* **30**, 2001165 (2020).
39. Feng, Y. et al. Ultrahigh-content Co–P cluster as a dual-atom-site electrocatalyst for accelerating polysulfides conversion in Li–S batteries. *Adv. Funct. Mater.* **32**, 2207579 (2022).
40. Zhao, C. et al. A high-energy and long-cycling lithium–sulfur pouch cell via a macroporous catalytic cathode with double-end binding sites. *Nat. Nanotechnol.* **16**, 166-173 (2021).
41. Su, L. et al. Cobalt-embedded hierarchically-porous hollow carbon microspheres as multifunctional confined reactors for high-loading Li-S batteries. *Nano Energy* **85**, 105981 (2021).
42. Hu, J., Zhang, L. Achieving F-doped porous hollow carbon nanospheres with ultrahigh pore volume via a gas–solid interface reaction. *J. Mater. Chem. A* **9**, 27560-27567 (2021).
43. Chen, G. et al. MOFs-derived porous Mo_2C–C nano-octahedrons enable high-performance lithium–sulfur batteries. *Energy Stor. Mater.* **25**, 547-554 (2020).
44. Xu, J. et al. MOF-derived porous N–Co_3O_4@N–C nanododecahedra wrapped with reduced graphene oxide as a high capacity cathode for lithium–sulfur batteries. *J. Mater. Chem. A* **6**, 2797-2807 (2018).
45. Sun, T. et al. $NiMoO_4$ nanosheets anchored on N–S doped carbon clothes with hierarchical structure as a bidirectional catalyst toward accelerating polysulfides conversion for Li–S battery. *Adv. Funct. Mater.* **31**, 2101285 (2021).
46. Shi, Z. et al. Boosting dual-directional polysulfide electrocatalysis via bimetallic alloying for printable Li–S batteries. *Adv. Funct. Mater.* **31**, 2006798 (2021).
47. Liu, X. et al. A three-dimensional nitrogen-doped graphene framework decorated with an atomic layer deposited ultrathin V_2O_5 layer for lithium sulfur batteries with high sulfur loading. *J. Mater. Chem. A* **8**, 12106-12113 (2020).
48. Lu, Y. et al. Hypercrosslinked polymerization enabled N-doped carbon confined Fe_2O_3 facilitating Li polysulfides interface conversion for Li–S batteries. *Adv. Energy Mater.* **11**, 2101780 (2021).
49. Li, Y. et al. CoSe nanoparticle embedded B,N-codoped carbon nanotube array as a dual-functional host for a high-performance Li-S full battery. *ACS Nano* **16**, 17008-17020 (2022).
50. Xiao, K. et al. Improving polysulfides adsorption and redox kinetics by the Co_4N nanoparticle/N-doped carbon composites for lithium-sulfur batteries. *Small* **15**, 1901454 (2019).
51. Wei, Y. et al. Rational design of multifunctional integrated host configuration with lithiophilicity-sulfiphilicity toward high-performance Li–S full batteries. *Adv. Funct. Mater.* **31**,

2006033 (2021).
52. He, J., Manthiram, A. 3D CoSe@C aerogel as a host for dendrite-free lithium-metal anode and efficient sulfur cathode in Li–S full cells. *Adv. Energy Mater.* **10**, 2002654 (2020).
53. Benítez, A. et al. Physical activation of graphene: An effective, simple and clean procedure for obtaining microporous graphene for high-performance Li/S batteries. *Nano Res.* **12**, 759-766 (2019).
54. Xing, Z., Li, G., Sy, S., Chen, Z. Recessed deposition of TiN into N-doped carbon as a cathode host for superior Li-S batteries performance. *Nano Energy* **54**, 1-9 (2018).
55. Huang, L. et al. Multifunctional hyphae carbon powering lithium–sulfur batteries. *Adv. Mater.* **34**, 2107415 (2022).
56. Yao, Y. et al. A dual-functional conductive framework embedded with TiN-VN heterostructures for highly efficient polysulfide and lithium regulation toward stable Li–S full batteries. *Adv. Mater.* **32**, 1905658 (2020).
57. Wang, P. et al. Dual-functional MgO nanocrystals satisfying both polysulfides and Li regulation toward advanced lithium−sulfur full batteries. *Small* **17**, 2103744 (2021).
58. Zhou, C. et al. Pulverizing Fe_2O_3 nanoparticles for developing Fe_3C/N-codoped carbon nanoboxes with multiple polysulfide anchoring and converting activity in Li-S batteries. *Adv. Funct. Mater.* **31**, 2011249 (2021).
59. Sun, W. et al. Monodispersed FeS_2 electrocatalyst anchored to nitrogen-doped carbon host for lithium–sulfur batteries. *Adv. Funct. Mater.* **32**, 2205471 (2022).
60. Li, H. et al. Dual-function, tunable, nitrogen-doped carbon for high-performance Li metal–sulfur full cell. *Small* **15**, 1804609 (2019).
61. Wu, S. et al. Engineering multi-chambered carbon nanospheres@carbon as efficient sulfur hosts for lithium–sulfur batteries. *J. Mater. Chem. A* **6**, 10891-10897 (2018).
62. Zhang, N. et al. Sulfur encapsulation by MOF-derived CoS_2 embedded in carbon hosts for high-performance Li–S batteries. *J. Mater. Chem. A* **7**, 21128-21139 (2019).
63. Liu, Y. et al. Constructing patch-Ni-shelled Pt@Ni nanoparticles within confined nanoreactors for catalytic oxidation of insoluble polysulfides in Li-S batteries. *Small* **15**, 1902431 (2019).
64. Nguyen, V. P. et al. Porous carbon textile decorated with VC/V_2O_{3-x} hybrid nanoparticles: Dual-functional host for flexible Li-S full batteries. *Energy Stor. Mater.* **46**, 542-552 (2022).
65. Li, S. et al. Implanting single-atom N_2-Fe-B_2 catalytic sites in carbon hosts to stabilize high-loading and lean-electrolyte lithium-sulfur batteries. *Energy Stor. Mater.* **55**, 94-104 (2023).
66. Zou, K. et al. A highly efficient sulfur host enabled by nitrogen/oxygen dual-doped honeycomb-like carbon for advanced lithium–sulfur batteries. *Small* **18**, 2107380 (2022).
67. Li, H. et al. A high-loading and cycle-stable solid-phase conversion sulfur cathode using edible fungus slag-derived microporous carbon as sulfur host. *Nano Res.* **16**, 8360-8367 (2023).
68. Pei, H. et al. Self-supporting carbon nanofibers with Ni-single-atoms and uniformly dispersed Ni-nanoparticles as scalable multifunctional hosts for high energy density lithium-sulfur batteries. *Small* **18**, 2202037 (2022).
69. Zhang, X.-Q., He, B., Li, W.-C., Lu, A.-H. Hollow carbon nanofibers with dynamic adjustable pore sizes and closed ends as hosts for high-rate lithium-sulfur battery cathodes. *Nano Res.* **11**,

1238-1246 (2018).
70. Chen, H. et al. 3D printing CO_2-activated carbon nanotubes host to promote sulfur loading for high areal capacity lithium-sulfur batteries. *Nano Res.* **16**, 8281-8289 (2023).
71. Zhang, W. et al. Co_4N-decorated 3D wood-derived carbon host enables enhanced cathodic electrocatalysis and homogeneous lithium deposition for lithium–sulfur full cells. *Small* **18**, 2105664 (2022).
72. Lin, C. et al. Porous nitrogen-doped carbon/MnO coaxial nanotubes as an efficient sulfur host for lithium sulfur batteries. *Nano Res.* **12**, 205-210 (2019).
73. Liu, S. et al. Superhierarchical cobalt-embedded nitrogen-doped porous carbon nanosheets as two-in-one hosts for high-performance lithium–sulfur batteries. *Adv. Mater.* **30**, 1706895 (2018).
74. Liu, R. et al. TiO_2 and Co nanoparticle-decorated carbon polyhedra as efficient sulfur host for high-performance lithium–sulfur batteries. *Small* **15**, 1804533 (2019).
75. Chen, K. et al. Metal–organic frameworks (MOFs)-derived nitrogen-doped porous carbon anchored on graphene with multifunctional effects for lithium–sulfur batteries. *Adv. Funct. Mater.* **28**, 1707592 (2018).
76. Lu, H. et al. Simultaneous growth of carbon nanotubes on inner/outer surfaces of porous polyhedra: Advanced sulfur hosts for lithium-sulfur batteries. *Nano Res.* **11**, 6155-6166 (2018).
77. Guo, D. et al. Single Mo–N_4 atomic sites anchored on N-doped carbon nanoflowers as sulfur host with multiple immobilization and catalytic effects for high-performance lithium–sulfur batteries. *Adv. Funct. Mater.* **32**, 2204458 (2022).
78. Jia, L. et al. High areal capacity flexible sulfur cathode based on multi-functionalized super-aligned carbon nanotubes. *Nano Res.* **12**, 1105-1113 (2019).
79. Guo, D. et al. Ni_3S_2 anchored to N/S co-doped reduced graphene oxide with highly pleated structure as a sulfur host for lithium–sulfur batteries. *J. Mater. Chem. A* **8**, 3834-3844 (2020).
80. Yao, W. et al. "Pea-pod-like" nitrogen-doped hollow porous carbon cathode hosts decorated with polar titanium dioxide nanocrystals as efficient polysulfide reservoirs for advanced lithium–sulfur batteries. *J. Mater. Chem. A* **6**, 18191-18205 (2018).
81. Zhang, H. et al. Nanopore-confined g-C_3N_4 nanodots in N, S co-doped hollow porous carbon with boosted capacity for lithium–sulfur batteries. *J. Mater. Chem. A* **6**, 7133-7141 (2018).
82. Ogoke, O. et al. Large-diameter and heteroatom-doped graphene nanotubes decorated with transition metals as carbon hosts for lithium–sulfur batteries. *J. Mater. Chem. A* **7**, 13389-13399 (2019).
83. Seo, S.-D., Yu, S., Park, S., Kim, D.-W. In situ conversion of metal–organic frameworks into VO_2–V_3S_4 heterocatalyst embedded layered porous carbon as an "all-in-one" host for lithium–sulfur batteries. *Small* **16**, 2004806 (2020).
84. Sun, W. et al. Catalytic Co_9S_8 decorated carbon nanoboxes as efficient cathode host for long-life lithium-sulfur batteries. *Nano Res.* **13**, 2143-2148 (2020).
85. Guan, L. et al. Intrinsic defect-rich hierarchically porous carbon architectures enabling enhanced capture and catalytic conversion of polysulfides. *ACS Nano* **14**, 6222-6231 (2020).
86. Deng, W., Xu, Z., Deng, Z., Wang, X. Enhanced polysulfide regulation via honeycomb-like carbon with catalytic MoC for lithium–sulfur batteries. *J. Mater. Chem. A* **9**, 21760-21770 (2021).

87. Weng, W. et al. Molten salt electrochemical modulation of iron–carbon–nitrogen for lithium–sulfur batteries. *Angew. Chem., Int. Ed.* **60**, 24905-24909 (2021).
88. Qian, J. et al. Enhanced electrochemical kinetics with highly dispersed conductive and electrocatalytic mediators for lithium–sulfur batteries. *Adv. Mater.* **33**, 2100810 (2021).
89. Wu, Q. et al. Integrated reactor architecture of conductive network and catalytic nodes to accelerate polysulfide conversion for durable and high-loading Li-S batteries. *Energy Stor. Mater.* **55**, 73-83 (2023).
90. Zhao, W. et al. Engineering metal-sulfides with cations-tunable metal-oxides electrocatalysts with promoted catalytic conversion for robust ions-storage capability. *Energy Stor. Mater.* **45**, 1183-1200 (2022).
91. Hussain, S. et al. Robust TiN nanoparticles polysulfide anchor for Li–S storage and diffusion pathways using first principle calculations. *Chem. Eng. J.* **391**, 123595 (2020).
92. Han, Z. et al. Machine-learning-assisted design of a binary descriptor to decipher electronic and structural effects on sulfur reduction kinetics. *Nat. Catal.* **6**, 1073-1086 (2023).
